# Risk of Liver Fibrosis in Methotrexate-Treated Patients: A Systematic Review

**DOI:** 10.7759/cureus.30910

**Published:** 2022-10-31

**Authors:** Sumahitha Bichenapally, Vahe Khachatryan, Asmaa Muazzam, Chandani Hamal, Lakshmi Sai Deepak Reddy Velugoti, Godfrey Tabowei, Greeshma N Gaddipati, Maria Mukhtar, Mohammed J Alzubaidee, Raga Sruthi Dwarampudi, Sheena Mathew, Safeera Khan

**Affiliations:** 1 Internal Medicine, California Institute of Behavioral Neurosciences & Psychology, Fairfield, USA; 2 Pathology Research, California Institute of Behavioral Neurosciences & Psychology, Fairfield, USA; 3 Research, California Institute of Behavioral Neurosciences & Psychology, Fairfield, USA; 4 Pediatrics, California Institute of Behavioral Neurosciences & Psychology, Fairfield, USA

**Keywords:** liver enzymes, methotrexate, hepatic cirrhosis, liver cirrhosis, liver fibrosis

## Abstract

Methotrexate (MTX), an antifolate agent, is recommended as the first-line disease-modifying antirheumatic drug (DMARD). In this systematic review, our goals were to assess liver fibrosis in methotrexate-treated patients, evaluate liver fibrosis in relation to treatment duration and cumulative dose, and identify differences based on the underlying disease. We followed the Preferred Reporting Items for Systematic Reviews and Meta-Analyses (PRISMA) guidelines to perform the systematic review. We thoroughly searched PubMed, PubMed Central (PMC), and Cochrane library databases to identify relevant articles based on predefined selection criteria. Studies were selected based on the following predefined eligibility criteria: English language, papers from the last 20 years, systematic reviews, observational studies, randomized controlled trials (RCTs), and clinical trials, which included papers on MTX playing roles in the development of liver fibrosis with the derived data transferred to a template. Following that, quality was assessed using the appropriate assessment tool for each study. The initial search yielded 512 results. Following a thorough review, 10 studies were chosen for final consideration: eight observational studies and two systematic reviews. Liver enzyme (LE) elevations during MTX therapy are a common but transient problem. Serial abnormal LE tests may be associated with liver pathology, but fibrosis development is uncommon. However, it is unclear from the literature how therapy should be adjusted in the case of elevated LE and to what extent MTX is linked to liver toxicity; definitive conclusions cannot be drawn because more research is needed.

## Introduction and background

Methotrexate (MTX) is a versatile immunosuppressant that is frequently used in the treatment of a variety of malignant, inflammatory, and autoimmune diseases. The European League Against Rheumatism and the American College of Rheumatology both recommend it as the first-line disease-modifying antirheumatic drug (DMARD) in the treatment of rheumatoid arthritis (RA) [1-2]. Since its first use in cancer in the 1950s, methotrexate has been proposed at lower doses in inflammatory disorders such as psoriasis, rheumatoid arthritis, and Crohn's disease [3-5]. Despite the fact that the efficacy and tolerability profiles of MTX are well established, liver toxicity has always been a concern [6-8].

In several psoriasis cohorts from the 1970s, patients treated with MTX had a prevalence of liver cirrhosis of up to 26% [9-10]. Furthermore, these studies suggested that higher cumulative MTX doses were linked to liver toxicity and fibrosis [11]. This served as the foundation for the Dermatology Society's recommendation to perform surveillance liver biopsies (LBs) at baseline and every 1.5 g of MTX [11-12]. The American College of Rheumatology (ACR) guidelines from 1994 only recommend an LB at baseline in RA patients with pre-existing risk factors for liver pathology and an LB during MTX therapy in the case of persistently elevated liver enzymes (LE) such as aspartate aminotransferase (AST) or decreased albumin, as studies suggested a link with liver pathology [13].

Clinical practice varies greatly as a result of these differences. In particular, the management of psoriatic arthritis (PsA), which is frequently treated by both specialties, can be challenging because the risk of liver fibrosis/cirrhosis in this subset of psoriatic patients is unknown. In conclusion, more agreement on monitoring liver toxicity during MTX therapy is needed, including the need for multiple LE tests, LB, and subsequent treatment adjustments/management. This comprehensive review aims to better understand the effect of methotrexate on liver toxicity.

## Review

Methods

Following our screening section, we report our method and results for systematic review using the Preferred Reporting Items for Systematic Reviews and Meta-Analysis (PRISMA) criteria and principles [14].

Search Sources and Strategy 

We searched electronic databases PubMed, PubMed Central (PMC), and the Cochrane library for articles based on Medical Subject Headings (MeSH) and keywords to identify the most relevant reviews and studies for analysis. "Liver Cirrhosis" and "Methotrexate" were among the keywords. For previously listed keywords, the MeSH strategy used in PubMed and PMC was the following: ("Liver Cirrhosis"[Majr] AND "Methotrexate/adverse effects"[Majr] OR "Methotrexate/poisoning"[Majr] OR "Methotrexate/toxicity"[Majr]). We assembled the keywords for a PubMed algorithm using the Boolean method. The articles were screened for relevance to the search question and selected based on inclusion and exclusion criteria.

Eligibility Criteria

The following criteria were used to select the literature papers for the systematic review:

Inclusion criteria: Papers published within the last 20 years. All articles chosen are peer-reviewed, relevant to the questions, and published in English. The population, intervention, comparison, and outcomes (PICO) model was used to determine eligibility.

Exclusion criteria: Papers discussing the pediatric and geriatric populations, as well as unpublished and gray literature, were excluded.

Articles Screening and Assessment for Eligibility

Duplicates were identified and removed. The remaining papers were then screened based on their titles and abstracts to eliminate those that were unqualified. The remaining data were then sorted using a set of inclusion and exclusion criteria. The remaining articles were thoroughly read and checked for quality. The review included only relevant papers that met more than 60% of the assessment criteria in the quality appraisal.

Risk Assessment Bias

The quality of the included studies was assessed using the tools shown, and only articles meeting >60% of the appraisal parameters were included, as shown in Table 1.

**Table 1:** Quality assessment using the preferred tools JBI tool: Joanna Briggs Institute (JBI) check tool; AMSTAR: Assessment of Multiple Systematic Reviews.

Type of studies	Tool used	Number of studies
Observational studies	JBI tool	8
Systematic review	AMSTAR checklist	2

Quality Appraisal of Studies

There are two types of studies included in the systematic review: cohort studies and cross-sectional studies. The Joanna Briggs Institute (JBI) check tool was used to assess the quality of these studies, and the National Institutes of Health (NIH) quality assessment tool was used to assess the quality of the cohort studies. Table 2 summarizes the findings.

**Table 2:** Quality assessment using the Joanna Briggs Institute (JBI) check tool for observational studies N/A: non-applicable.

Study/Year of publication	Were the two groups similar and recruited from the same population?	Were the exposures measured similarly to assign people to both exposed and unexposed groups?	Was the exposure measured in a valid and reliable way?	Were confounding factors identified?	Were the strategies to deal with confounding factors stated?	Were the groups/participants free of the outcome at the start of the study (or at the time of exposure)?	Were the outcomes measured in a valid and reliable way?	Was the follow-up time reported sufficient to be long enough for outcomes to occur?	Was the follow-up complete, and if not, were the reasons to loss to follow-up described and explored?	Were strategies to address incomplete follow-up utilized?	Was appropriate statistical analysis used?
Lindsay et al. 2009 [15]	N/A	Yes	Yes	Yes	Yes	Yes	Yes	Yes	N/A	Yes	Yes
Bafna et al. 2021 [16]	N/A	Yes	Yes	Yes	Yes	Yes	Yes	Yes	Yes	Yes	Yes
Kim et al. 2015 [17]	Yes	Yes	Yes	N/A	Yes	Yes	Yes	Yes	Yes	N/A	Yes
Park et al. 2010 [18]	Yes	Yes	Yes	Yes	Yes	Yes	Yes	Yes	Yes	N/A	Yes
Barbero-Villares et al. 2011 [19]	N/A	Yes	Yes	Yes	Yes	Yes	Yes	N/A	N/A	Yes	Yes
Lahdenne et al. 2002 [20]	Yes	Yes	Yes	Yes	Yes	Yes	Yes	Yes	Yes	No	Yes
Laharie et al. 2006 [21]	N/A	N/A	Yes	Yes	Yes	Yes	Yes	Yes	Yes	Yes	Yes
Laharie et al. 2010 [22]	Yes	Yes	N/A	Yes	Yes	Yes	Yes	Yes	Yes	Yes	Yes

Results

Search Outcome 

**Figure 1:**
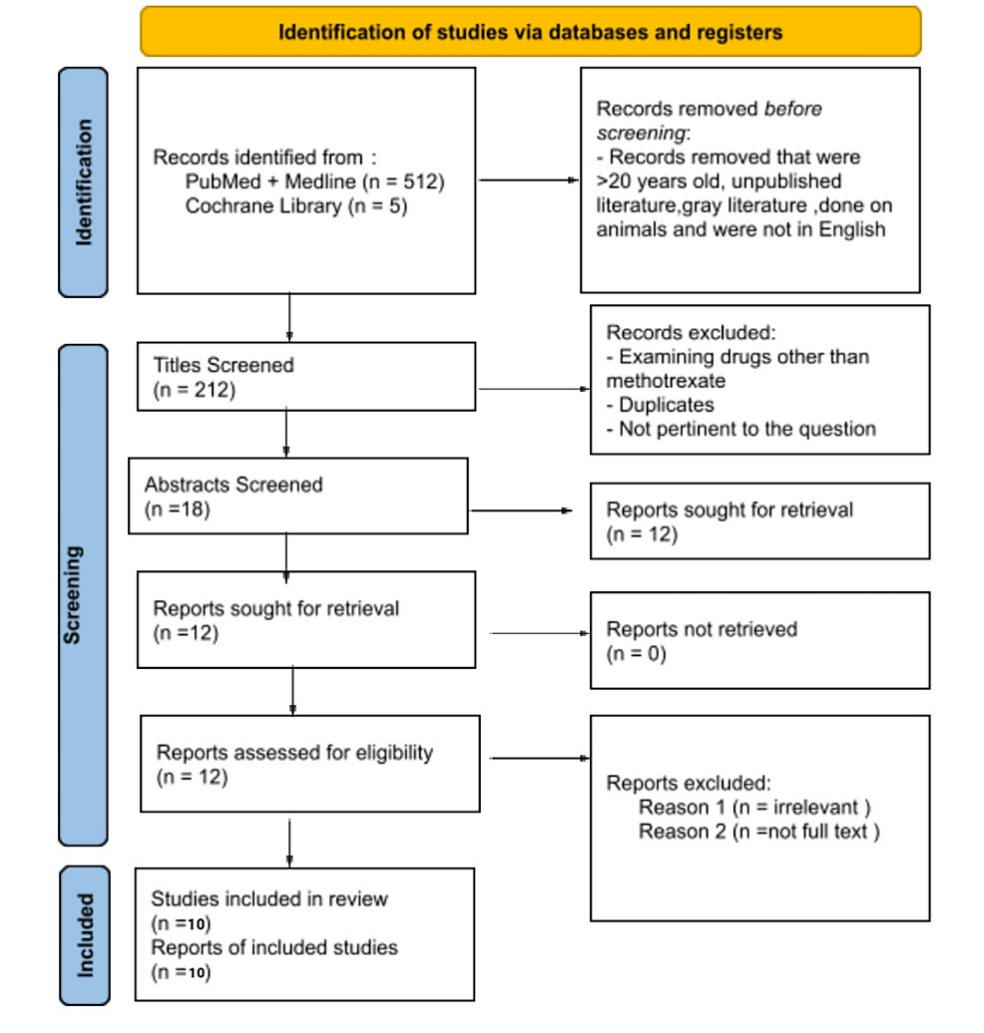
The article filtering processes are depicted in the PRISMA flow diagram PRISMA: Preferred Reporting Items for Systematic Reviews and Meta-Analysis.

A total of 517 papers were identified using the MeSH and keywords through four databases, yielding 212 results after removing 305 duplicates. The results were initially screened based on the eligibility criteria (inclusion and exclusion), followed by the title and abstract, yielding a total of 12 articles. Only 10 articles were used after a quality assurance check. Two systematic reviews and eight observational studies were among the 10 articles. The effects of methotrexate on the liver in patients with chronic inflammatory diseases were discussed in these articles. Figure 1 shows the PRISMA flow diagram of the article filtering process [14].

Actual Results 

Our systematic review gathered information from limited data on patients treated with methotrexate for chronic inflammatory diseases (RA, psoriasis, PsA, and Crohn's). This includes prospective analysis as well as self-reports from patients. While all of the articles chosen shared the common goal of researching the relationship between methotrexate and hepatic fibrosis, the number of participants, demographics, and study criteria varied. Table 3 summarizes the pertinent information from each of the 10 published papers. This review paper includes information from 1,626 participants.

**Table 3:** The effects of methotrexate on liver fibrosis in chronic inflammatory disease patients MTX: methotrexate; AST: aspartate aminotransferase; ALT: alanine aminotransferase; RA: rheumatoid arthritis; SWE: shear wave elastography; JIA: juvenile idiopathic arthritis; DMARD: disease-modifying antirheumatic drug; LE: liver enzyme; LB: liver biopsy; BMI: basal metabolic index.

Author and year of publication	Purpose of the study	Number of patients/procedures used	Type of study	Conclusion
Lindsay et al. 2009 [15]	Assessment of liver fibrosis in patients with psoriasis and psoriatic arthritis on long-term, high cumulative dose MTX therapy	54/liver biopsies	Prospective study	There was no correlation found between MTX dosing, duration, cumulative dose, or disease duration and hepatic fibrosis.
Bafna et al. 2021 [16]	Prevalence of liver fibrosis in rheumatoid arthritis patients on long-term MTX therapy	75/FibroScan	Cross-sectional	Long-term MTX use in RA patients was linked to increased liver stiffness. Obesity and increased waist circumference were common risk factors in patients with liver fibrosis. There was a significant correlation between higher cumulative MTX dose and FibroScan scores.
Kim et al. 2015 [17]	To investigate concerns about liver toxicities, including liver fibrosis, induced by long-term use of MTX in RA patients	185/Real-time shear wave elastography (SWE)	Prospective study	Significant liver fibrosis on SWE was observed in approximately 5% (9 out of 185 MTX-treated RA patients) and was associated with only a high BMI but not with the cumulative MTX dose (4,825 mg), implying that other comorbidities may play a more important role in liver fibrosis.
Park et al. 2010 [18]	To assess the degree of liver fibrosis with transient elastography and noninvasive biochemical methods in rheumatoid arthritis patients treated with methotrexate	177	Prospective study	In rheumatoid arthritis patients treated with a high cumulative dose of methotrexate, significant liver fibrosis is rare and is not accurately detected in patients with abnormal liver enzymes. The kilopascal values and levels of biochemical markers are not related with the cumulative dose of methotrexate but are related with the AST to ALT ratio, AST to platelet ratio index, and haptoglobin level.
Barbero-Villares et al. 2011 [19]	Evaluation of liver fibrosis in methotrexate (MTX)-treated patients by transient elastography	53/FibroScan	Prospective study	No correlation could be observed between liver stiffness and cumulative dose of MTX.
Lahdenne et al. 2002 [20]	Hepatotoxicity in patients with juvenile idiopathic arthritis (JIA) receiving long-term methotrexate therapy	36	Prospective study	Treatment of JIA with MTX at 20-30 mg/m^2^ in combination with disease-modifying antirheumatic drugs (DMARDs) and corticosteroids may contribute to minor, reversible liver abnormalities.
Laharie et al. 2006 [21]	To evaluate liver fibrosis with FibroScan and non-invasive biochemical methods in Crohn’s disease patients treated with methotrexate	54/FibroScan and liver biopsies	Prospective study	Significant liver fibrosis is rare in Crohn's disease patients treated with a high dose of methotrexate and is not accurately detected with liver enzyme abnormalities.
Laharie et al. 2010 [22]	Assessment of liver fibrosis with transient elastography and FibroTest in patients treated with methotrexate for chronic inflammatory diseases	518/FibroTest	Case-control study	Severe liver fibrosis is a rare occurrence in MTX patients and is unrelated to the total or cumulative dose.
Visser and van der Heijde 2009 [23]	To systematically review the literature on liver toxicity in rheumatoid arthritis (RA) and psoriatic arthritis (PsA) patients treated with methotrexate (MTX), as an evidence base for generating clinical practice recommendations for the management of MTX and the indication for a liver biopsy (LB) in case of elevated liver enzymes (LEs)	47	Systematic review	According to this review, LE elevations during MTX therapy are a common but transient problem, that serial abnormal LE tests may be associated with liver pathology, but that cirrhosis/fibrosis caused by MTX is uncommon.
Maybury et al. 2014 [24]	To provide relevant data on methotrexate and liver fibrosis in people with psoriasis	429	Systematic review	According to liver biopsy data, the data report/findings from this paper show an association between MTX use and disease progression.

Discussion 

The Mechanism of Methotrexate

A cross-sectional study conducted by Bafna et al. reported that the mechanism of MTX-induced hepatotoxicity is due to oxidative stress-mediated abnormal activation of hepatic "Ito" cells, which leads to collagen deposition in the perisinusoidal extracellular matrix, and the risk increases with cumulative MTX dose, whereas a prospective study conducted by Kim et al. and Park et al. reported that the effects of many MTX toxicities are caused by inhibiting purine and pyrimidine synthesis by blocking assorted enzymes, and responses from this blockage are accountable for many toxicities such as bone marrow suppression, stomatitis, and hepatotoxicity [16-18]. The precise mechanism by which low-dose MTX modulates inflammation in RA is unknown; however, the following mechanisms are associated with anti-inflammatory and immunosuppressive effects: increased intracellular and extracellular adenosine release, decreased proinflammatory cytokine release, decreased neutrophil chemotaxis, and decreased angiogenesis [18].

Effects of Liver Fibrosis

According to Park et al. prospective study, rheumatoid arthritis is a systemic autoimmune disease with an unknown cause that is characterized by chronic polyarticular inflammation and can lead to irreversible joint damage, disability, and deformity [18]. Corticosteroids and DMARDs, particularly MTX, are commonly used to treat rheumatoid arthritis. The use of MTX for the treatment of early and institutional RA is strongly advised. The elevation of liver enzymes is the second most common adverse effect (AE) of MTX use, after gastrointestinal upset. According to a recent study, 769 patients receiving MTX monotherapy had at least one episode of elevated liver enzymes. About 20.2% of patients had elevations up to twice the upper limit of normal, and 3.7% stopped taking MTX due to hepatotoxicity. Cirrhosis is not clinically significant in rheumatoid arthritis patients receiving a high cumulative dose of MTX. Hepatic dysfunction risk factors are well managed. Transient elastography (TE) ensures patient compliance and safety and can be a useful tool for screening high-risk populations to identify patients due to its superiority. Using TE should be researched further in the future to provide definitive values. Patients with hepatic impairment and various rheumatic diseases should be considered [18].

Barbero-Villares et al. conducted a prospective study [19]. They investigated some limitations in cases of obesity and ascites; it had a relatively high rate of specialized failures when compared to other studies. Those cases in which liver stiffness could not be assessed had an average basal metabolic index (BMI) of 33.6 kg/m^2^, and the significant degree of obesity significantly influenced the results. Individual treatment opinions can be obtained using transient elastography (TE) without the need for frequent liver biopsies. Transient elastography (TE) is widely permitted and accepted in clinical settings. Its properties indicate that it will become a more useful investigation in clinical practice, particularly in therapeutic studies and when patients are reluctant to consent to one or more repeat liver biopsies. When prescribed in standard doses, MTX does not cause significant liver fibrosis, with no significant differences between patients with rheumatoid arthritis, inflammatory bowel disease (IBD), or psoriasis. Furthermore, FibroScan appears to be useful for assessing and long-term monitoring of patients with liver cirrhosis who are being treated with MTX for chronic inflammatory diseases [19].

A prospective study by Lindsay et al. [15] reports that studies that act toward serial liver biopsies in psoriasis serve with long-term MTX have an occurrence of hepatic fibrosis of 13%-34% and cirrhosis of 0%-20%. Their group of long-term MTX-treated patients was chosen for psoriasis arthritis (PsA; n=47) and psoriasis (n=7¼) under regular outpatient follow-up with other risk factors for hepatotoxicity [15]. Sixteen (30%) had insulin based on diabetes, obesity, or both. Eight (15%) still drink above the suggested weekly quantity of alcohol in spite of advice to the contrary, and six (11%) admitted to having a history of drinking. In spite of this, their study shows a prevalence of mild and medically insignificant fibrosis of 20% and no clinical disease or cirrhosis. The specialist only stopped using MTX for a single patient with persistently elevated liver function tests and mild liver fibrosis. Although the sampling error in a liver biopsy is up to 33%, it is unlikely that it resulted in the absence of cirrhosis in their group. Microanatomy features were almost like announced results from patients with psoriasis who have not had MTX. This raises questions about whether cases of non-alcoholic steatohepatitis (NASH) or alcoholic cirrhosis are to blame for reports of MTX liver toxicity that date back much further. In our study, no link can be put together between MTX dosing, progressive dose, or disease duration and hepatic fibrosis, confirming other studies. According to the American College of Rheumatology (ACR) guidelines for long-term MTX monitoring, histological presence is safe for preventing cirrhosis in PsA, as evidenced by published results from patients with psoriasis who have not received MTX. If the liver function test (LFT) is normal, then sequential liver biopsies do not seem to be specified.

A cross-sectional study by Bafna et al. [16] found an increased prevalence of cirrhosis on transient elastic imaging in RA patients on long-term MTX and a big connection with the progressive dose of MTX. When accessible, FibroScan follow-up was recorded for patients with cirrhosis and compared to basic clinical characteristics and FibroScan scores. Proposed procedures of MTX-induced hepatotoxicity comprise oxidative stress and unusual liver activation. Ito cells cause collagen deposition within the pyrogenic region extracellular matrix, mainly at higher cumulative MTX doses. The generality of cirrhosis and liver fibrosis in patients with psoriasis/psoriatic arthritis taking MTX ranges from 2% to 23% or 0% to 4%, respectively [16].

In a prospective study by Lahdenne et al. [20], the consensus is that only liver biopsies do not support long-term MTX treatment or the risk of severe hepatotoxicity from low-dose MTX in juvenile idiopathic arthritis (JIA). However, this study also shows that the treatment of juvenile idiopathic arthritis with high doses of MTX with DMARD and corticosteroids might contribute to portal inflammation and steatosis of the liver, the changes that are feasible risk factors for liver cirrhosis. More practicable studies require to ascertain appropriate guidelines for monitoring patients with JIA receiving aggressive therapy treatment with antirheumatic drugs [20].

In a prospective study by Kim et al. [17] known to be an efficient treatment for RA, amid the side effects of MTX, the elevation of liver enzymes is the second most typical AE after gastrointestinal upset. This study was designed and conducted prospectively to handle concerns about liver toxicity, including liver fibrosis that will arise from continuing use of MTX and faltering issues with risk factors apart from MTX. One of the foremost important findings of this study was even after using a relatively high cumulative dose of 4,825 mg and a treatment duration of 330 weeks, cirrhosis of the liver greater than 8.6 kPa which is estimated as hepatic sclerosis was found in precisely nine (4.9%) of the 185 patients with autoimmune disease. In summary, this study showed that the large liver fibrosis, determined by a liver stiffness score greater than 8.6 kPa on shear wave elastography (SWE) encounter in approximately 5% of RA patients, and prolonged MTX treatment has only been related to a better basal metabolic index (BMI) but not with a cumulative MTX dose. To assess the progression of fibrosis, SWE may be a common, simple, risk-free, and accurate detection tool for liver cirrhosis and is clinically important and useful to acknowledge candidates for liver biopsy in high-risk patients. Future large, multicenter studies are warranted [17].

In a prospective study by Laharie et al. [21] on Crohn's disease, methotrexate may be a recommended steroid-sparing and maintenance therapy. However, its use is restricted due to toxicity, which ends up in discontinuation in 10%-18% of patients. Early gastrointestinal side effects (nausea, vomiting, diarrhea, stomatitis), likewise as hematological toxicity, will be prevented by folinic acid supplementation without reducing efficacy. During the first use of this drug, abnormal liver function tests were noted with alarming frequency. Lastly, significant cirrhosis of the Crohn's disease (CD) is rare in patients treated with high doses of methotrexate. A vibrating scan may be a reliable and noninvasive method to identify liver fibrosis which will be recommended for the patients. A liver biopsy can only be performed to diagnose concomitant liver diseases (such as alcohol disease, non-alcoholic fatty liver disease (NAFLD), and primary sclerosing cholangitis) and in patients with high FibroScan values or with persistent liver function test (LFT) abnormalities; more future studies must confirm these preliminary data [21].

A case-control study by Laharie et al. [22] is the largest study evaluating liver fibrosis in patients treated with methotrexate for various infections. We found that neither a high cumulative dose nor longer treatment duration was related to higher alanine aminotransferase (ALT) level FibroScan and FibroTest values. Since methotrexate was introduced into treatment, there are more feared long-term side effects like cirrhosis. Corresponding in keeping with studies from the 1980s, cirrhosis of the liver is widespread at 0%-33% and cirrhosis at 0%-26%, and it is estimated to be more common in psoriasis than in autoimmune disorders such as Crohn's disease. However, in addition to recent studies published after characterizing hepatitis C virus (HCV) infection and NASH, the incidence of MTX-induced cirrhosis was perceived to be lower (0%-6%), mainly in psoriasis patients, suggesting a possible overestimate in earlier series. This prevalence should be compared to the estimated 0.8% prevalence of cirrhosis within the general population. Lastly, severe cirrhosis is rare in treated patients within the case of MTX; it absolutely was certainly overestimated in past studies. Noninvasive screening methods and limitations of liver biopsy should be considered in patients with elevated FibroScan, FibroTest results, or other common risk factors, irrespective of methotrexate treatment regimen, dose, or duration. Further prospective longitudinal studies are imperative to verify this approach, including studies evaluating the cost-effectiveness of this recommendation [22].

Systematic review studies by Visser and van der Heijde [23] and Maybury et al. [24] summarize connected evidence available on MTX liver toxicity in RA and PsA. Inside the boundaries of what was available, the results are taken under consideration within the literature crafting discussions within the 3E initiative resulting in the event of recommendations for clinical practice to administer MTX treatment [23-24]. There was a 22% increased risk of "any fibrosis" on biopsy after MTX use. There was a trend toward MTX use and progression to associated significant fibrosis, but this was not statistically significant. Of note, the cumulative and duration of MTX treatment were not related to liver fibrosis/cirrhosis at biopsy (as independent variables). From these data, we will conclude that the employment of MTX increased the cause of fibrosis within the study group; however, we should always qualify for the following: MTX increases the chance of fibrosis, but not in everyone and not alone. The shortage of a transparent dose-dependent relationship indicates that other factors can influence the event of the pathology.

Limitations

This systematic review consisted of 10 published articles of literature, which has limitations. One such limitation was that only two systematic reviews were sourced from our three databases; this might be partly because of our eligibility criteria, which focused on literature published in English within the past 20 years and focused on adults older than 18 years old. As a result, several papers may are excluded just because they did not meet the inclusion criteria. There were also differences in the number of subjects between papers like Kim et al. [17], which had a sample size of 185 participants, compared to Lahdenne et al. [20], which had only 34 patients. The individual limitation of every study regarding study length, sample size, and drug effects made it difficult to facilitate adequate comparisons.

## Conclusions

Our objective was to see the consequences of MTX on cirrhosis in patients treated with MTX. Lastly, this review shows that hepatotoxicity may be a relatively minor concern during MTX treatment in inflammatory diseases. Abnormal liver enzymes are also related to disease, but there is no evidence that changing treatment if liver enzymes are elevated is critical to forecasting all future diseases. However, based on the results of the collected literature, it appears that there has been a decision. Few clinical studies were found examining whether MTX use significantly affected hepatotoxicity in MTX-treated patients. While the data gathered from various surveys and studies offered some insight, the results were conflicting. Given the low prevalence of cirrhosis and also the risk of complications, only liver biopsy seems justified as a definitive method of histological confirmation of disease. Therefore, no definitive conclusions are often drawn at this point. Ideally, more research is required to research this question further and specialize in the long-term effects of MTX use and objective assessment of elevated liver enzyme levels using hospital visitation records.
